# 

**Published:** 1897-07-10

**Authors:** 


### EARLY EDITION.

**Notices of Births, Deaths, and Marriages** are inserted in this column at a charge of 2s. 6d. for an announcement not exceeding four lines.

**DEATH.**

Bowen.—June 17th, 1897, at 167, Strand Road, Bootle, Liverpool, Winifred, late Nurse at Victoria Hospital, Burnley, and The Hospital, Blackpool, aged 30 years. (255)

### NOTICE TO CORRESPONDENTS.

All MS., Letters, Books for Review, and other matters intended for the Editor should be addressed The Editor, "The Hospital," The Hospital Building, 28 & 29, Southampton Street, Strand, W.C.

The Editor cannot undertake to return rejected MS., even when accompanied by stamped directed envelope.

**Notice to Advertisers and Subscribers.**

All Advertisements, Orders for Copies of The Hospital, and Business Communications should be addressed to **THE MANAGER, (Not to The Editor),** The Hospital, The Hospital Building, 28 & 29, Southampton Street, Strand, London, W.C.

The Cost of Subscription, post free, is as follows:—Per week, 2½d.; per quarter, 2s. 8d.; per half-year, 5s. 3d.; per annum, 10s. 6d. Foreign:—Per annum, 15s. 2d.; payable, in all cases, in advance.

### Scraps and Gleanings.

The Hospital of St. Cross, at Rugby, is to be enlarged.

It is proposed to build a new hospital at Omagh for County Tyrone.

A home for incurables, with 60 beds, is to be erected at Dundee. £50,000 is asked for.

It has been decided to amalgamate the Abergavenny Dispensary with the Cottage Hospital.

A hospital for seamen is to be built at Cardiff in place of the "Hamadryad" hospital ship.

The establishment of a convalescent home for Middlesbrough, to be situated at Ayton, has been proposed.

The total expenditure at the Queenstown General Hospital in 1896 was £581. 171 patients were treated, as against 167 last year.

The new infectious diseases hospital for Edinburgh, to be erected at Colinton Mains, is to cost a quarter of a million.

The extension proposed to be made at the Sunderland and North Durham Eye Infirmary will increase its accommodation from 18 beds to 30.

A suggestion has been made that the North Shields and Tynemouth Dispensary shall be amalgamated with the Tynemouth Victoria Jubilee Infirmary.

At the recent annual meeting of the Victoria Hospital for Sick Children, Hull, the smallness of the annual subscription list was commented on.

About 500 more cases were treated in 1896 than in 1895 at the Nottingham and Midland Eye Infirmary, the exact numbers being 3,813 in 1896 and 3,296 in 1895.

During 1896 447 in-patients (112 more than in 1895), and 2,990 out-patients (a decrease of 58), were treated at the Royal Alexandra Hospital for Sick Children, Brighton

The Hospital Saturday Collection in 1897 in aid of the Belfast Royal Infirmary realised £300, or about £50 more than in 1896, and about the same amount as that collected in 1895.

It has been proposed that street collections in connection with the Hospital Saturday Fund at Wolverhampton should be abolished, but considerately the matter has been deferred.

The managers of the Aberdeen Ophthalmic Institution the other day declined to agree to the suggestion made by the Royal Infirmary that the two institutions should be amalgamated.

The gross income of the Norfolk and Norwich Hospital for the year 1896 was £9,092, or £1,129 in excess of the previous year, a difference more than accounted for by an increase of £740 in legacies and £556 in donations. The gross expenditure was £9,071, against £8,550 in 1895. 1,558 in and 5,765 out patients were treated during the year, an increase of 84 and 1,234 respectively.

Three hundred and forty-five persons were admitted to the in-patient department of the Birmingham and Midland Hospital for Women during 1896, the total number of operations being 333, of which 200 involved abdominal section. Three thousand one hundred and ten out-patients were also treated. Of the 3,514 persons who applied for relief during the year, 202 cases were rejected as being unsuitable.

At Lady Day last, the end of the financial year, there was a debit balance on the accounts of the Devon and Exeter Hospital of £1,966. The income amounted to £7,306, and the expenditure to £8,862.

On an average last year each patient at the Glasgow Ear Hospital was seen nearly six times. One thousand two hundred and ninety-two new patients were treated, of whom 66 were treated in the wards.

No less than 8,610 accidents (or 24 a day) were brought to the North Charitable Infirmary, Cork, in 1896. The total number of in-patients treated was 855, and the out-patients made 23,267 attendances.

The average number of patients treated annually at the Hereford General Infirmary for the years 1890 to 1896 inclusive was in-patients 685 and out-patients 2,730. In 1896 694 in and 3,143 out-patients were treated.

Though the Seacombe Cottage Hospital contains only 12 beds the daily average number occupied in 1896 was 14½. The average cost per in-patient was £3 9s., against £3 5s. 6d. in 1895. A new central hospital to cost £8,000 is proposed.

The report of the North Ormesby Cottage Hospital states that the council have not been able, owing to lack of funds, to make the needful alterations and additions to the kitchen and domestic offices of the hospital, nor to proceed with the isolation ward.

The receipt of legacies amounting to £530 has wiped out the debt on the Brighton Hospital for Women, and left a balance in hand of £77. In-patients in 1896 numbered 85, as compared with 38 in 1895; out-patients, 765 against 928; and maternity cases attended at the patients' homes, 1,024 against 1,172.

The receipts of the Glasgow Hospital Sunday Fund for 1896 show an advance of nearly £274 on the preceding twelve months. 332 churches and 204 Sunday schools contributed. The total amount collected was £4,343, of which £4,102 was distributed amongst the hospitals for which the collections are made, viz., the Royal, Western, and Victoria Infirmaries.

Cottage hospitals are to be established at the following places as memorials of the Diamond Jubilee: Duns, N.B., Colne (Lancs), Ramsbottom (Lancs), Kingston (Surrey), Skibbereen, Aston, Colwyn Bay, Kingussie (Invernesshire), Ashbourne, Woking, Clacton, Chippenham, Worksop, Torrington (Devon), Tonypandy (Glamorganshire), Bishop Auckland, Broseley, and Tonbridge. Proposals to erect cottage hospitals at the following places were not carried: Wednesbury, Moffat, and Wendover (Bucks).

The Carlisle Hospital Sunday Committee have adopted the following amended method of distributing its funds amongst the hospitals to whom grants are made: The fund available for the purpose shall, in the first instance, be allocated to a grant of 3d. per head for each patient who has been on the books of each institution in the last year for which a printed report is available; and the balance remaining after this capitation grant shall be allocated to the beneficiary institutions according to their requirements, taking for the principal basis of distribution the respective expenditures after the deduction of all income arising from investments, interest, dividends, &c., and collections paid direct to the institutions, workmen's contributions being considered as collections paid direct to the institutions.

### The Student of Medicine.

### APPOINTMENTS.

J. Philip, F.R.C S.,Eng., L.R.C.P.,Lond., appointed Official Railway Medical Officer for the Wellington Section of the New Zealand Government Railways. J. S. Pickford, M.R.C.S.Eng., L.R.C.P, appointed Medical Officer of Health to the Little Lever Urban District. S. J. Roberts, M.R.C.S.Eng., L.R.C.P.Lond., appointed Medical Officer for the No. 3 District of the Aston Union. A. Rotherham, M.A., M.B., B.C.Cantab, appointed Assistant Medical Officer at the London County Asylum, Cane Hill, Purley, Surrey. R. H. Russell, F.R.C.S.Eng., appointed Surgeon to In-patients at the Melbourne Hospital for Sick Children, Australia. S. W. Scott, M.B., B.S., appointed House Physician to the Sunderland Infirmary. G. A. Shackel, M.R.C.S.Eng., L.R.C.P.-Lond., appointed Medical Officer of Health to the Ludlow Rural District Council. W. A. Skinner, M.B., C.M.Edin., appointed Junior Assistant Medical Officer to the Royal Asylum, Sunnyside, Montrose. G. W. B. Slader, L.S.A., L.R.C.S.I., appointed Medical Officer for the Blyton District of the Gainsborough Union. Dr. Smith, appointed Medical Officer for the Murragh Dispensary District. L. Smith, M.R.C.S., L.R.C.P., appointed Resident Medical Officer to the Westminster General Dispensary, Soho. W. A. Smyth, L.R.C.P , L.R.C.S.Edin., appointed Medical Officer for the Ashtead District of the Epsom Union. P. E. Snoad, M.R.C.S.Eng., L.R C.P.Lond., appointed Medical Officer for the Sixth District of the Leicester Union. Dr. Stenhouse, appointed Medical Officer for the Arnold District of the Basford Union. L. W. Stephens, M.R.C.S.Eng., L.S.A., appointed Medical Officer for the Workhouse and the First District of the Westbourne Union. Dr. C. Stewart, appointed Medical Officer for the Parish of Kilmaurs. H. Stott, M.R.C.S.-Eng., L.R.C.P.Lond., D.P.H.R.C.S Eng., appointed Medical Officer of Health to the Battle, Cuckfield, Eastbourne, East Grinstead, Hailsham, Newhaven, Ticehurst, and Uckfield Rural District Councils, and Cuckfield, Hayward's Heath, and Uckfield Urban District Councils. A. Thorp, M.R.C S.Eng., L.R C.P.-Lond., appointed Medical Officer to the Wooldale District of the Huddersfield Union. N. Townsend, L.R.C.P., L.R.C.S.I., appointed Extern Physician to the South Charitable Infirmary, Cork. J. D. Wardale, M.B.Durh., appointed Surgical Registrar to the Royal Infirmary, Newcastle-on-Tyne. W. B. Warrington, M.D.Lond., appointed Honorary Assistant Physician to the Hospital for Consumption and Diseases of the Chest, Liverpool. Dr. Wigham, appointed Medical Officer of Health to the South Molton Town Council. W. M. Willis, F.R.C.S.Eng., appointed House Surgeon to the Sunderland Infirmary. R. C. Bartlett, M.R.C.S.Eng., L.R.C.P., appointed Medical Officer of Health to the Romsey Rural District Council. R. G. Burton, M.D., L.R.C.S.Edin., appointed Medical Officer of Health to the Greenwood Urban District. E. Clapham, M.D.Edin , M R.C.S., appointed Medical Officer to the Wimbledon Isolation Hospital. J. M. Coates, M.B., C.M.Edin., appointed Assistant House Surgeon to the Northern Hospital, Liverpool. P. R. Dennehy, L.K.Q.C.P.I., L.R.C.S.Edin., appointed Medical Officer for the Lismore Dispensary District. J. Eaton, M.D.Glasg., M.B., C.M., appointed Medical Officer for the Cleaton District of the Whitehaven Union. W. McAdam Eccles, M.S.Lond., F.R.C.S.Eng., appointed Examiner in Anatomy to the Society of Apothecaries of London. Dr. H. M. Edwards, appointed Medical Officer for the Markyate District of the Luton Union. T. E. Gee, F.R.C.P. Edin., F.R.C.S.Edin., L.S.A., appointed Honorary Anæsthetist to the Hospital for Epilepsy and Paralysis, Regent's Park. Mr. W. R. Gibson, appointed Assistant Medical Officer to the St. Saviour's Union Infirmary. G. Grindlay, M D.Edin., appointed Medical Officer for the Kingswinsford District of the Stourbridge Union. H. E. W. Hoffmeister, B.A.Camb., M.R.C.S., appointed Medical Officer of Health to the East Cowes Urban District Council. R. Lake, F.R.C.S., appointed Surgeon Laryngologist to the North London Hospital for Consumption, Hampstead.

